# A *Bacillus anthracis* Genome Sequence from the Sverdlovsk 1979 Autopsy Specimens

**DOI:** 10.1128/mBio.01501-16

**Published:** 2016-09-27

**Authors:** Jason W. Sahl, Talima Pearson, Richard Okinaka, James M. Schupp, John D. Gillece, Hannah Heaton, Dawn Birdsell, Crystal Hepp, Viacheslav Fofanov, Ramón Noseda, Antonio Fasanella, Alex Hoffmaster, David M. Wagner, Paul Keim

**Affiliations:** a Center for Microbial Genetics and Genomics, Northern Arizona University, Flagstaff, Arizona, USA; b Division of Pathogen Genomics, The Translational Genomics Research Institute, Flagstaff, Arizona, USA; c School of Informatics, Computing and Cyber Systems, Northern Arizona University, Flagstaff, Arizona, USA; d Laboratorio Azul, Azul, Buenos Aires. Argentina; e Istituto Zooprofilattico Sperimentale of Puglia and Basilicata, Foggia, Italy; f Centers for Disease Control and Prevention, Atlanta, Georgia, USA

## Abstract

Anthrax is a zoonotic disease that occurs naturally in wild and domestic animals but has been used by both state-sponsored programs and terrorists as a biological weapon. A Soviet industrial production facility in Sverdlovsk, USSR, proved deficient in 1979 when a plume of spores was accidentally released and resulted in one of the largest known human anthrax outbreaks. In order to understand this outbreak and others, we generated a *Bacillus anthracis* population genetic database based upon whole-genome analysis to identify all single-nucleotide polymorphisms (SNPs) across a reference genome. Phylogenetic analysis has defined three major clades (A, B, and C), B and C being relatively rare compared to A. The A clade has numerous subclades, including a major polytomy named the trans-Eurasian (TEA) group. The TEA radiation is a dominant evolutionary feature of *B. anthracis*, with many contemporary populations having resulted from a large spatial dispersal of spores from a single source. Two autopsy specimens from the Sverdlovsk outbreak were deep sequenced to produce draft *B. anthracis* genomes. This allowed the phylogenetic placement of the Sverdlovsk strain into a clade with two Asian live vaccine strains, including the Russian Tsiankovskii strain. The genome was examined for evidence of drug resistance manipulation or other genetic engineering, but none was found. The Soviet Sverdlovsk strain genome is consistent with a wild-type strain from Russia that had no evidence of genetic manipulation during its industrial production. This work provides insights into the world’s largest biological weapons program and provides an extensive *B. anthracis* phylogenetic reference.

## IMPORTANCE

The 1979 Russian anthrax outbreak resulted from an industrial accident at the Soviet anthrax spore production facility in the city of Sverdlovsk. Deep genomic sequencing of two autopsy specimens generated a draft genome and phylogenetic placement of the Soviet Sverdlovsk anthrax strain. While it is known that Soviet scientists had genetically manipulated *Bacillus anthracis* with the potential to evade vaccine prophylaxis and antibiotic therapeutics, there was no genomic evidence of this from the Sverdlovsk production strain genome. The whole-genome SNP genotype of the Sverdlovsk strain was used to precisely identify it and its close relatives in the context of an extensive global *B. anthracis* strain collection. This genomic identity can now be used for forensic tracking of this weapons material on a global scale and for future anthrax investigations.

## INTRODUCTION

Anthrax is a zoonotic disease caused by *Bacillus anthracis* with a relatively small impact on global human health, but it has become notorious and widely feared due to its use and potential as a biological weapon. In its spore form, the bacterium represents a highly stable quiescent entity that is capable of surviving for decades, a critical part of its ecology, global distribution, evolution, and infectivity. The vegetative phase allows for cellular proliferation following spore germination in a host animal. The vegetative form expresses specific mechanisms for avoiding the innate host immunity, with some of these encoded on two large virulence plasmids—pXO1 and pXO2 (1). Adaptive immunity can be highly effective at preventing disease, and interestingly, anthrax was the first bacterial disease mitigated with a vaccine (2). Vaccine development for this pathogen is an important veterinary and public health measure, but research with a potential weapon of mass destruction (WMD) unfortunately can also lead to highly similar research supporting pathogen weaponization. Therefore, the treaty created by the Biological Weapons Convention of 1975 with 175 State Parties prohibited all offensive efforts with any biological agent, including anthrax (3).

The *B. anthracis* spore’s stability, potential for aerosolization, and its ability to cause acute pulmonary disease have historically led to multiple nations weaponizing this bacterium. It is well documented that large-scale production of spores was accomplished by the United States, the United Kingdom, and the Soviet Union (4). Industrial spore production involves numerous quality control features to ensure spore stabilization, particle size, and the retention of virulence with extensive growth. These state-sponsored programs were to cease with the Biological Weapons Convention of 1975. However, there are least two recent examples of anthrax spores being used in biological attacks: the Aum Shinrikyo cult’s attempted liquid dispersal of *B. anthracis* in 1993 (5) and the 2001 United States anthrax letters that killed five and sickened an additional 17 (6).

The offensive anthrax weapons development programs were stopped in the United States and United Kingdom in the 1960s but continued covertly in the Soviet Union for at least another 20 years (4). Soviet, and later Russian, research on anthrax included projects to genetically modify *B. anthracis* strains. First, antibiotic resistance was genetically engineered into the vaccine strain STI-1 using recombinant DNA and a plasmid vector (7). This effort resulted in multidrug resistance to penicillin, rifampin, tetracycline, chloramphenicol, macrolides, and lincomycin with retention of normal colony morphology (7). The stated goal of this research was the development of novel vaccines that allowed the simultaneous use of a live vaccine strain and antibiotics in the case of human exposure. Without the drug-resistant live vaccine strain, long-term antibiotic therapy is required. Second, the program genetically engineered hemolytic properties from *Bacillus cereus* into *B. anthracis* by the transfer of cereolysin AB genes into the STI-1 strain, again via a recombinant plasmid (8). This genetic change resulted in a strain with unique pathogenic features that could overcome the standard STI-1 vaccine protection in animal studies. The generation of a hemolytic *B. anthracis* strain was ostensibly for research purposes to understand basic host immunomodulation during anthrax, yet yielded a strain and strategy that could defeat vaccine protection. Manipulation of the *B. anthracis* genome to change its phenotypic properties can and has been accomplished, raising concerns about dual use.

Evidence of the Soviet anthrax program’s continuation and its scale were revealed by the 1979 industrial accident in Sverdlovsk, USSR (now known as Ekaterinburg), where at least 66 people died of inhalational anthrax (9). This event has been shrouded in mystery, with governmental denials and little public investigation, but it does represent one of the largest known human inhalational anthrax outbreaks in history (4). According to local sources (4, 10), in early April 1979 safety air filters were compromised during routine maintenance at the Ministry of Defense’s (MOD) Scientific Research Institute of Microbiology (SRIM) spore production facility, known as Compound 19. This resulted in a plume of spores that spread downwind and caused human anthrax cases up to 4 km away and animal cases up to 50 km away (9). Russian pathologists investigated these deaths and generated formalin-fixed tissues from multiple victims for analysis. These specimens showed evidence of anthrax (11) and along with later PCR-based DNA analyses (12–14) that detected *B. anthracis* confirmed that this cluster of deaths was indeed due to anthrax.

Here we have continued the Sverdlovsk anthrax investigation through deep sequencing of the formalin-fixed tissues from two of the victims to generate a draft genomic sequence of the infecting *B. anthracis* strain*.* In this article, we also report the phylogenetic analysis of single-nucleotide polymorphisms (SNPs) discovered among 193 whole-genome sequences, which provided a phylogenetic context for analysis of the Sverdlovsk samples and can be used for similar analysis of other samples of interest. This provides a high-resolution analysis with detailed clade and subclade structures defined by a curated SNP database. SNP genotyping accurately places the Sverdlovsk strain into a subclade defined by the Tsiankovskii vaccine strain. We also examine the genome sequences for evidence of genetic engineering and adaptation to large production biology. The results show the power of combining modern molecular biology methods with a high-resolution curated SNP database in order to analyze a *B. anthracis* strain involved in a historic anthrax incident.

## RESULTS

### A high-resolution reference phylogeny.

**FIG 1:**
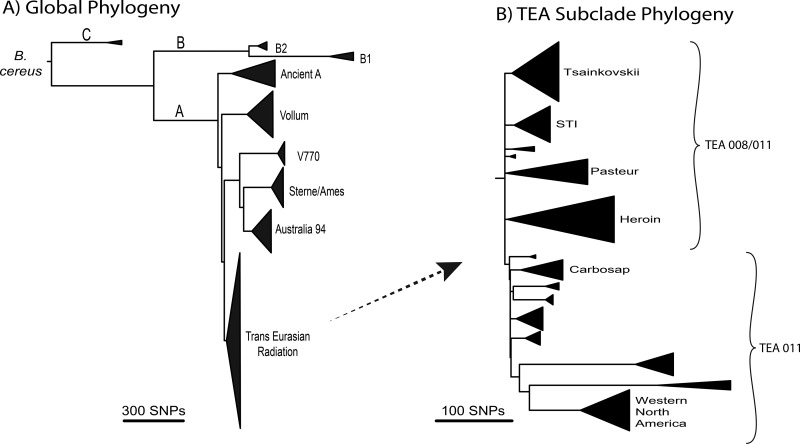
Phylogenetic structure of *B. anthracis*. Orthologous SNPs (11,989 total; 5,663 parsimony informative) from whole-genome sequences were analyzed by maximum parsimony to generate a phylogenetic tree. The major clades are collapsed in this figure, but the complete tree is available in the supplemental material (see Fig. S1). The overall consistency index is 0.98.

We have constructed a high-resolution reference phylogeny from a large global *B. anthracis* strain collection. This is presented with collapsed clades (Fig. 1) to illustrate the overall phylogenetic structure but with complete branching details and annotated SNPs in the supplemental material (see Fig. S2 and S3). The global phylogeny is comprised of genomes from 193 strains (see Table S1 in the supplemental material) that represent the global diversity as defined by other subtyping methods such as multilocus variable-number tandem-repeat analysis (MLVA) (15) and canonical SNPs (16–19). Genomic sequence comparisons yielded 11,989 SNPs (5,663 parsimony informative) from orthologous genomic segments (see Table S2 in the supplemental material). This represents an average of only 1 SNP every ~500 bp across the entire genome and breadth of this species. A list of SNPs that define each branch and the homoplastic SNPs is provided in Table S3 in the supplemental material to facilitate efforts by other researchers to place their strains in these established clades.

The deeper phylogenetic relationships (Fig. 1A) are consistent with those reported previously with a more limited number of genomes (16–18, 20–24) as well as across different phylogenetic methods (maximum likelihood using the general time reversible [GTR] model of evolution and neighbor joining). There are three major clades, C being basal to the A and B clades (Fig. 1A). Members of the A clade are most frequently observed across the globe (~90%), with B (~10%) and C (<1%) members being much less frequent (16). The A clade can be divided into four major monophyletic subclades, with the “Ancient A” group being basal to the other subclades (Fig. 1A). Members of the “TransEurAsia” (TEA) subclade are most commonly observed as they have been highly successful across large and diverse geographic areas (16).

The unusually short lengths of the deepest branches of the TEA clade, coupled with the high frequency of isolates and geographic expansion, are indicative of a rapid and extensive evolutionary radiation (Fig. 1B). Many sublineages of this clade diverged before mutations occurred, leading to a lack of synapomorphic characters (shared alleles that could group some of these sublineages together) and the existence of a large polytomy (a node with 7 immediate descendant lineages: Tsiankovskii, STI, Pasteur, Heroin, TEA 011, and two lineages with 1 and 2 genomes each). The expansion of each of these lineages also leads to multiple distinct groups, also often with very little topological resolution in the deeper nodes. Given the number of isolates assigned to the TEA 011 group, the TEA clade can be divided into two main subgroups: paraphyletic TEA 008/011 (A.Br.008/011) and monophyletic TEA (A.Br.011).

### Sverdlovsk specimen sequence analysis.

By direct DNA sequencing, we generated metagenomic data from paraffin-embedded formalin-fixed pathology specimens from two anthrax victims from the 1979 outbreak in Sverdlovsk, USSR. The presence of *B. anthracis* DNA in these specimens had been previously established (12), and targeted gene sequencing had also been successful (13, 14); however, until recent technological advances in DNA sequencing, this could only be accomplished by first PCR amplifying small portions of the genome. Sequencing across both the MiSeq and HiSeq Illumina platforms produced ~300 million reads and 20 Gb of nucleotide sequence data across both specimens. A direct mapping of reads against the finished genome of the Ames ancestor genome with BWA-MEM demonstrated that only 1.2% of the total sequence data mapped to the reference genome. This is expected as DNA is from human tissue. The *B. anthracis* coverage represented an average sequencing depth of 24× across the chromosome, with >100× coverage of pXO1 and pXO2 plasmids. These data covered 99% of the Ames ancestor genome, including both plasmids, with at least one read. Alignment stats are shown in Table 1.

**TABLE 1:** Alignment status for Sverdlovsk *B. anthracis* genomes

Library	Type	Length (bp)	No. of reads (pairs)			Coverage (×)			Chromosome breadth at 1× (%)
			Total	Trimmed	Mapped	Chromosome	pXO1	pXO2	
Svd-1	HiSeq	93	6.4E07	4.5E07	5.4E05	8	32	55	96
Svd-1	MiSeq	300	1.0E07	3.4E06	1.5E05	1	3	27	30
Svd-2	HiSeq	93	7.5E07	6.6E07	1.0E06	15	85	104	99
Svd-2	MiSeq	300	1.2E07	3.6E06	1.7E05	1	6	28	36
Combined data			1.6E08	1.2E08	1.9E06	25	126	214	99

From the reads, we assembled the Sverdlovsk genome into 128 contigs with an *N*_50_ size of 74 kb. A prediction of coding regions (CDSs) with Prodigal (25) on this assembly identified 5,579 CDSs; the same analysis on the Ames ancestor genome identified 5,756 CDSs. This demonstrates that while most of the genome was successfully assembled, parts of the genome may have been dropped from the assembly, most likely from insufficient coverage or collapsed repeats.

### Data quality of the Sverdlovsk *B. anthracis* genome.

Formalin fixation is known to damage nucleic acids, and this was demonstrated by the small size of the extracted DNA fragments (12), but its effect upon the validity of the Sverdlovsk genomic sequence was unknown. The intrinsic error rate in a sequencing project can be measured by mapping individual sequencing reads to a high-quality reference genome. This generates an estimate of the raw read error rate at each nucleotide and across the whole genome, representing a sequencing quality measurement particularly relevant to SNP identification. In a comparison of *B. anthracis* sequencing reads from Sverdlovsk pathology specimens to those from DNA isolated from culture, we observe a higher number of errors (see Fig. S1 in the supplemental material). The average rate per nucleotide was 0.2% for the culture-generated DNA versus 0.5% for the formalin-fixed tissue. In both cases, a true polymorphism would not be determined from a single read but rather from the consensus of multiple read coverage at any particular genomic position; however, see Sahl et al. (26) for a low-coverage SNP-calling strategy. We further examined the consequences of this differential error rate by searching for the conservation of known SNPs along a particular phylogenetic path within these genomes. These were identified in the 193-genome phylogeny (Fig. 1), independent of the Sverdlovsk genome. There were 329 known SNP changes along the branches that connect the Ames ancestor reference to the composite Sverdlovsk genome (Fig. 1; see Table S3 and Fig. S2, S3D, and S3G in the supplemental material). All 329 SNP sites were present in the composite genome assembly. Excluding 29 SNP sites on the pXO1 and pXO2 plasmids because they have higher copy numbers, the coverage per SNP averaged 20× at 273 of the remaining 300 genomic positions on the chromosome. Fourteen of the other chromosomal SNP sites contained less than 10 reads per site but still corresponded exactly to the expected base changes. Overall, we were able to discover and verify all of the known SNPs using the Sverdlovsk pathology specimen sequencing data. Based upon these two error estimations, we are confident that the sequenced genomes are of sufficient quality to justify our conclusions.

### Phylogenetic position of the Sverdlovsk strain.

Based upon shared SNPs, the Sverdlovsk genomes fall within the “Tsiankovskii” subclade of the TEA 008/011 group (Fig. 1B). Within this group, it is most closely related to two other Asian strains both of which are used as vaccines. There are only 13 SNPs on the branch to the Sverdlovsk genomes, 25 on the branch to Tsiankovskii, and 52 on the branch to Cvac02 (see Tables S2 to S4 in the supplemental material). These three genomes emerge from a polytomy, showing rapid divergence of these lineages before shared SNPs could arise. As this clade is comprised of laboratory strains, this divergence may be due to anthropogenic establishment of different lineages from a laboratory stock. Other clade members were isolated from anthrax-killed animals and are mostly Eastern European in origin, with the exception of one from China and one from Norway. Therefore, with the exception of the three “domesticated” strains, the clade members are naturally occurring wild-type strains.

### Sverdlovsk *B. anthracis* genome-specific SNPs.

The sequencing and analysis of Sverdlovsk genomes offer an opportunity to detect SNPs and to look for possible strain mixtures or contaminating DNA profiles from two of the tissue samples. To do this, nucleotides from individual reads are tabulated, and less than 100% agreement represents potential errors or mixtures at that genomic position. In particular, we are interested in the 13 SNPs that are unique to Sverdlovsk genomes as they allow a comparison to all other strains outside this group to identify mixtures. Table 2 shows the consensus read results from Sverdlovsk-specific SNPs, and overall there are only 7 variants, resulting in an error rate of 1.6%, which is only slightly higher than the overall error rate of 0.5%. In addition, we note that 6 of the 7 differences are located near the ends of reads, where the error rate is higher (data not presented). One SNP (NC_007530: 5138018) was detected between the two specimens, and this contrast appears to represent a real difference as it was supported by >18 reads. A small number of SNPs between these two specimens might be observed given the population size associated with large-scale production and subsequent amplification *in vivo*. Otherwise, we find no evidence in these two particular Sverdlovsk specimens for strain mixtures. It is important to recognize that these two specimens did not show mixed alleles at the *vrrA* locus analyzed by Jackson et al. (12).

**TABLE 2:** Read mixtures at Sverdlovsk genome-specific SNPs

SNP site	SNP	Read depth (×)	% of consensus	Genomic element
28576	G to A	78	97.2	pX02
59151	C to T	49	100	Chromosome
112138	A to G	36	100	Chromosome
602999	C to A	34	100	Chromosome
1359179	C to T	27	100	Chromosome
2138718	A to T	20	100	Chromosome
2979549	A to G	27	100	Chromosome
3593664	T to C	31	89.9	Chromosome
4034596	G to A	17	93.7	Chromosome
4236707	T to C	30	100	Chromosome
4504222	C to T	22	100	Chromosome
4896833	T to C	17	100	Chromosome
5186004	C to A	46	100	Chromosome

### Genetic engineering evidence.

**FIG 2:**
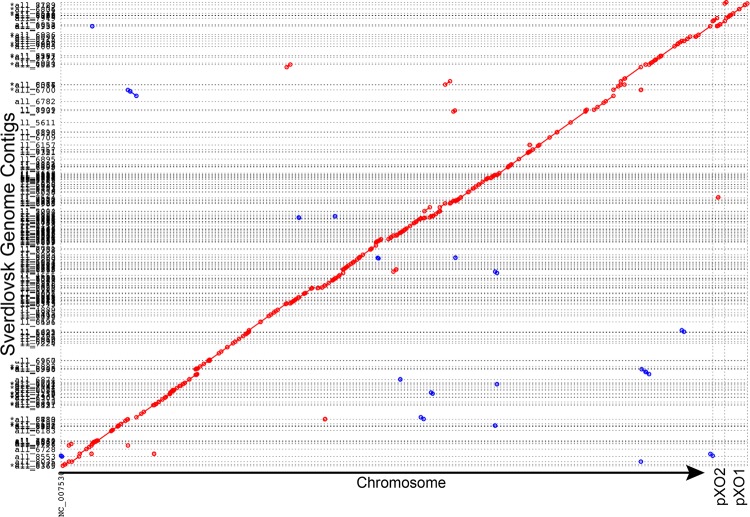
Sverdlovsk contigs aligned to the Ames ancestor genome. The reads from both autopsy specimens were combined for *de novo* assembly, which resulted in 128 contigs. These are aligned against the Ames ancestor chromosome and two plasmids, and the synteny was visualized with mummerplot. Greater than 99% of the Ames ancestor genome is represented by these 128 contigs.

Particular genes and SNP signatures in the Sverdlovsk genomes were examined for evidence of genetic manipulation of this strain. In the chromosome, fluoroquinolone resistance is known to be determined by amino acid changes in the *gyrA* and *parC* genes (27), rifampin resistance is associated with changes in the *rpoB* gene (28), and penicillin resistance is associated with changes in β-lactamase gene expression (29). With regard to amino acid changes in associated genes, the Sverdlovsk genomes contained wild-type drug-susceptible alleles. The cereolysin genes and plasmid sequences used by Russian scientists to alter *B. anthracis* phenotypes (7, 8) were not present. In addition, the read data were examined for other common genetic engineering vectors, which were not detected, from an alignment of raw reads against the NCBI UniVec database. The alignment of the 128 contigs to the Ames ancestor revealed no novel genes (Fig. 2), though this was not a closed genome. Hence, there is no evidence from this analysis of either molecular-based genetic engineering or classical bacteriological selection for altered drug resistance phenotypes.

## DISCUSSION

The *B. anthracis* global phylogeny is one of the most robust evolutionary reconstructions available for any species. This is possible because orthologous SNPs represent highly stable evolutionary characters with very low homoplasy, and their rarity in this genome precludes any effects from mutational saturation. This species’ evolutionary reconstruction is a function of its spore-vegetative cycle biology and, in particular, its ecological niche. The dormant spore stage is important for its dispersal and transmission, limiting evolutionary changes and restricting interactions with near-neighbor *Bacillus* species, making it resistant to horizontal gene transfer. Hence, the *B. anthracis* pan-genome is only slightly larger than the core genome, with variation primarily due to decay via gene deletion. Environmental growth outside the host is possible, but does not appear to represent a significant opportunity to shape this bacterium’s genome and evolution. Long quiescent periods in the spore phase may create a “time capsule” where few or no mutations are generated, resulting in a highly homogeneous pathogen. In this sense, its niche differs from its close relative *B. cereus*, which is environmentally adapted with occasional pathogenic replication in a host (30). Fortuitously, the genome variation that we can identify through whole-genome sequencing generates insights into anthrax history and allows predictions about its ecology.

The clade structure we observe with whole-genome sequencing is consistent with previous descriptions using lower-resolution methods or few genome sequences. What we add in this report is the precise definition of branching points, accurate branch length determinations, and the definition of canonical evolutionary characters for strain identification. Branch topology determination has been problematic with other molecular methods because of the abundance of short branches and polytomies at critical positions in the evolutionary structure. The A clade itself, but in particular its subclade TEA, is evidence for evolutionary radiations representing genetic bottlenecks, long-distance dispersal, and bursts in the fitness of these lineages. Even in a radiation, binary fission of replicating bacterial cells should result in phylogenetic structure that could be identified with sufficiently discriminatory methods. But in some cases, such as with the TEA clade, even whole-genome analysis does not yield topological phylogenetic structure, arguing for a very tight genetic expansion. This subclade contains a large portion of the world’s anthrax burden (16), making this radiation event seminal. Molecular clock analyses for 106 subroot dated isolates (see Table S1 and Fig. S4 in the supplemental material) and the 48 dated TEA isolates (see Fig. S5 in the supplemental material) have revealed a complete lack of temporal signal among members of this relatively contemporary data set, leaving the exact timing of this radiation dependent upon phylogeographic hypotheses. These models are controversial and vary widely in their temporal predictions (22, 31). To ensure that the lack of molecular clock signal is not due to error arising from various sequencing methods, we pruned the phylogeny to clade A isolates with sister taxa that have dates of isolation within 5 years of each other. We then removed all non-parsimony informative sites, such that only shared SNPs were used to reconstruct the phylogeny as we assume that sequencing errors are unlikely to occur on shared branches. As in the former root-to-tip analyses, a temporal signal was not evident (see Fig. S6 in the supplemental material). The lack of a consistent SNP substitution rate could be due to variation in the spore phase of the *B. anthracis* life cycle. However, other spore-forming bacteria have demonstrated temporal signal in their phylogenies (32), suggesting that specific ecological dynamics related to sporulation/infection rates must also be involved for anthrax. Ancient genomes from archeological sites would greatly assist in the temporal calibration of key branch points.

Detailed genome databases are a great resource for public health and forensic investigations of disease outbreaks (33). As disease events occur, they allow for the real-time matching of similar types and source identification. But pathogens are dynamic, and databases must be continually updated with isolates from contemporary outbreaks. For some pathogens, a few months can allow for genomic divergence that will make source tracking problematic (34, 35). The availability of high-quality reference databases sets the stage for further sampling (23). It is important to define the relevant subpopulation for additional investigative sampling (36), and this will not be possible prior to a disease outbreak.

Inspired by other preserved pathology tissue DNA analyses (37), two *B. anthracis* genome sequences from victims of the Soviet military accident in Sverdlovsk, USSR, were generated by deep sequencing of formalin-fixed autopsy specimens. Although only ~1.2% of the sequenced reads were associated with the pathogen, enough information was obtained for high-resolution phylogenetics and for draft genome assemblies. A higher than normal error rate was observed in the Sverdlovsk samples, likely due to the nature of the specimen preservation, but sufficient depth of coverage was still obtained to accurately genotype known SNP loci and to identify strain-specific polymorphisms. Contigs assembled from the reads are syntenic with reference genomes and consistent with isolates from natural anthrax outbreaks, with no extraneous reads associated with cloning vectors or novel toxins. Additionally, there was no evidence of *B. anthracis* strain mixtures in these two particular specimens. Jackson et al. (12) reported mixed alleles at the *vrrA* locus for some tissue samples, but not the two analyzed in this report. The *vrrA* locus could not be assembled from these specimens due to its repeat structure, and the other victim specimens had very limited DNA that was prohibitive for metagenomic analysis. Hence, our analysis does not eliminate the possibility that mixed strains were involved in the Sverdlovsk anthrax outbreak.

The Soviet “battle strain” 836 was isolated from nature (10) and used for industrial spore production in the 1960s and 1970s, which was mostly prior to the advent of recombinant DNA methods. Traditional selection for mutants resistant to antibiotic resistance was certainly possible prior to 1979, but no such mutations are evident in the Sverdlovsk strain genomes. The great similarity of the genomes to other natural isolates argues for minimal laboratory manipulation. It is well established that *B. anthracis* attenuates with laboratory culturing, and selection for drug resistance frequently has secondary phenotypic consequences that would not be desirable for a weapons strain (27). All of this is highly suggestive of a weapons program that identified a suitable strain, maintained master cell stocks to avoid extensive passage, and performed minimal manipulations in order to maintain virulence. This strategy must have been used to produce large quantities of highly virulent material, as evidenced by the anthrax deaths in 1979.

## MATERIALS AND METHODS

### Sverdlovsk specimen DNA sequencing.

DNA was extracted from paraffin-embedded formalin-fixed tissues from two victims as previously described (12). These extracts were characterized by quantitative PCR (qPCR) (13), and the two samples (Svd-1, 7.RA93.15.15, spleen; Svd-2, 21.RA93.38.4, lymph node) with the lowest threshold cycle (*C_T_*) values were subjected to Illumina sequencing, first on a MiSeq and later on a HiSeq 2000. Sequencing libraries were constructed using the standard Kapa Biosystems Illumina NGS Library reagent kit (catalog no. KK8232; Kapa Biosystems, Boston, MA), using 12 cycles in the final amplification reaction. Due to the highly degraded nature of the input DNA, fragment size selection prior to library preparation targeted fragments that were <500 bp. Both samples yielded libraries with enough material for sequencing and were pooled and then sequenced using an entire MiSeq 600 cycle paired-end run with V3 chemistry. This same pool was subsequently sequenced on a HiSeq 2000, using two lanes.

### Sequence analysis.

Sequencing adapters were trimmed from reads with Trimmomatic (38). For SNP discovery, reads were aligned against the finished genome of the Ames ancestor (NC_007530, NC_007322, and NC_007323) with BWA-MEM (39), and SNPs were called with the UnifiedGenotyper method in GATK (40, 41). These methods were wrapped by the NASP pipeline (http://tgennorth.github.io/NASP/) (42). Functional information was applied to SNPs with SnpEff (43).

### Error profile analysis.

To understand the error profiles in the Sverdlovsk genomes, reads were aligned against the Ames ancestor with BWA-MEM, and for each position, the number of alleles that conflicted with the dominant allele was divided by the total number of bases at the position; this value was considered the per-base error rate. As a control, this procedure was also performed for another genome (A0362) in the same phylogenetic group. Error rates were binned into different categories and represented as a histogram (see Fig. S1 in the supplemental material).

### Genome assembly.

To obtain a draft genome assembly, reads from both victims were combined and assembled with SPAdes v.3.6.0 (44). The first 200 bases of each contig were aligned against the GenBank (45) nucleotide database with BLASTN (46) to identify contigs not associated with *B. anthracis*; contigs that significantly aligned against human sequence were removed from the assembly. The contiguity of the assembly was then improved through a reference-guided approach with AlignGraph (47), using the Ames ancestor as the reference. The assembly was polished with Pilon v.1.3.0 (48), resulting in 128 contigs. A dot plot analysis using mummerplot (49) was used to examine the synteny against the Ames ancestor as the reference.

### Phylogenetic reconstructions.

**FIG 3:**
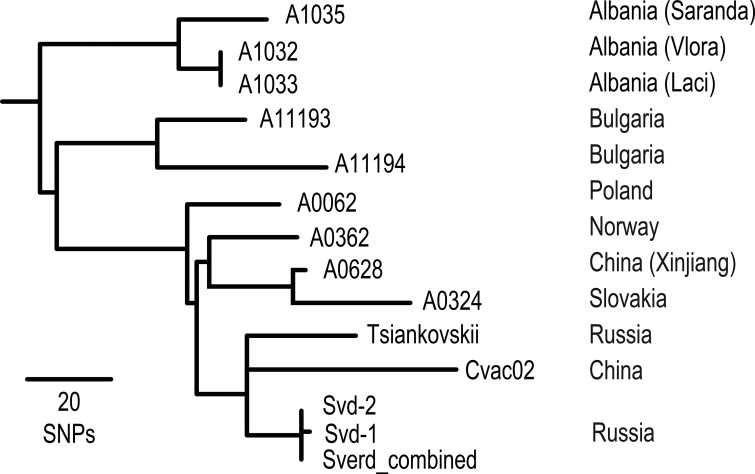
The Tsiankovskii clade. A phylogenetic tree of the closest relatives to the Sverdlovsk genomes is shown. One SNP was discovered between Svd-1 and Svd-2. The Sverd_combined genotype is identical to Svd-2.

We compared the genomes of 193 strains of *B. anthracis* (see Table S1 in the supplemental material) against the Ames ancestor to find SNPs (see Table S2 in the supplemental material) using the *in silico* Genotyper (50) and the Northern Arizona SNP Pipeline (42). All SNP loci—even those that are missing in some of the genomes—were retained for phylogenetic analyses. We used parsimony criteria and a heuristic search with default options using PAUP 4.0b10 (51) to infer phylogenetic trees. We report homoplasy using the consistency index as a measure of accuracy (52) as bootstrapping is a poor measurement of accuracy for trees with little homoplasy (53) in clonal organisms (20, 21). It should be noted, however, that the consistency index is influenced by the number of taxa, impacting direct comparisons across trees. The phylogeny for all *B. anthracis* genomes was rooted according to Pearson et al. (21). Trees of individual clades and subclades were rooted using a *B. anthracis* strain from another clade or the first strain to diverge from the rest of the group as determined by the overall phylogeny of *B. anthracis*. Phylogenetic branches were named according to precedent (16) and designated on trees (Fig. 3; see Fig. S2 and S3 in the supplemental material). In short, each branch contains a prefix “A.Br,” “B.Br,” “A/B.Br,” or “C.Br,” depending on the major clade designation, followed by an assigned number based upon the order of branch discovery within each of the major clades. This method maintains the branch name from previous publications and allows for the identification of novel branches. However, branch numbers of adjacent branch numbers will often not be contiguous. For each SNP, the branches on which character state changes occurred, as determined by PAUP (51) using the DescribeTrees command, is listed in the supplemental material (see Table S3 in the supplemental material).

For evolutionarily stable characters such as SNPs found in clonal organisms like *B. anthracis*, a single locus can define a branch and thus serve as a “canonical SNP” (16, 20, 21, 54). As such, the character states of only a small number of SNP loci need to be interrogated in order to place an unknown strain into the established phylogenetic order. The list of SNPs on each branch (see Table S3) thus serves as a resource of signatures that can be used to define a branch. However, new genome sequences will cause existing branches to be split, requiring additional branch names and updating the branch designation of these SNPs.

### Accession number(s).

All reads were submitted to the NCBI Sequence Read Archive for 21.RA93.38.4 (SRR2968141 and SRR2968216) and 7.RA93.15.15 (SRR2968143 and SRR2968198).

## SUPPLEMENTAL MATERIAL

Figure S1  Read error rate profile across the genome for Sverd and a culture DNA (A0362: SRR2968203). Reads were aligned to the Ames ancestor, and the compositions of base calls were compared. Error rates were determined by dividing the number of minor allele calls by the total number of calls. The error rates were then binned into categories from no error to total error. The frequency of calls in each bin is represented by the height of histograms. The results demonstrate that while both genomes had low error rates, the Sverdlovsk genome had a higher error profile than a contemporary, pure culture. Download Figure S1, PDF file, 0.3 MB

Figure S2  Maximum parsimony phylogeny of 193 *B. anthracis* genomes. Consistency index (CI [excluding parsimony uninformative characters]) = 0.9657. Names of major branches are indicated in blue text. Branch names within each clade are included in supplemental figure 3 with separate panels dedicated to each clade. Download Figure S2, PDF file, 0.5 MB

Figure S3  (A) Maximum parsimony phylogeny of the “Ancient A” clade. The formal name for this clade is A.Br.006/005. This clade currently contains 14 genomes and 904 SNPs. Consistency index (CI [excluding parsimony uninformative characters]) = 0.9931. Names of branches are indicated in blue text. (B) Maximum parsimony phylogeny of the “Vollum” clade. The formal name for this clade is A.Br.005/010. This clade currently contains 22 genomes and 1,446 SNPs. CI (excluding parsimony uninformative characters) = 0.9910. Names of branches are indicated in blue text. (C) Maximum parsimony phylogeny of the “V770” clade. The formal name for this clade is A.Br.004/003. This clade currently contains 13 genomes and 384 SNPs. CI (excluding parsimony uninformative characters) = 1.0. Names of branches are indicated in blue text. (D) Maximum parsimony phylogeny of the “Sterne/Ames” clade. The formal name for this clade is A.Br.003/014. This clade currently contains 21 genomes and 818 SNPs. CI (excluding parsimony uninformative characters) = 0.9823. Names of branches are indicated in blue text. (E) Maximum parsimony phylogeny of the “Australia94” clade. The formal name for this clade is A.Br.003/002. This clade currently contains 22 genomes and 1,158 SNPs. CI (excluding parsimony uninformative characters) = 0.9892. Names of branches are indicated in blue text. (F) Phylogeny of the “TEA” clade. This clade contains many large subclades that are presented in detail in panels G-I. Names of major branches are indicated in blue text. (G) Maximum parsimony phylogeny of the “Tsiankovskii” subclade (Fig. 3). This subclade is part of the “TEA” clade and is within the A.Br.008/011 clade. This subclade currently contains 14 genomes and 375 SNPs. CI (excluding parsimony uninformative characters) = 0.9921. Names of branches and branch lengths are indicated in blue text. (H) Maximum parsimony phylogeny of the “Heroin” subclade. This subclade is part of the “TEA” clade and is within the A.Br.008/011 clade. This subclade currently contains 14 genomes and 1,392 SNPs. CI (excluding parsimony uninformative characters) = 1.0. Names of branches are indicated in blue text. (I) Phylogeny of the “TEA 011” subclade. This subclade is part of the “TEA” clade. This clade contains the “WNA” subclade, which is presented in detail in panel J. This subclade currently contains 40 genomes and 1,835 SNPs. CI (excluding parsimony uninformative characters) = 0.9712. Names of branches are indicated in blue text. (J) Phylogeny of the “WNA” subclade. This subclade is part of the “TEA” clade. This subclade currently contains 10 genomes and 343 SNPs. CI (excluding parsimony uninformative characters) = 1.0. Names of major branches are indicated in blue text. Download Figure S3, PDF file, 0.4 MB

Figure S4  Molecular clock analysis for all genomes with isolation dates, except for the three C-branch isolates (2002013094, A1055, and 2000031052). (A) Linear regression analysis of root-to-tip distances extracted by Tempest (55) from a neighbor-joining tree reconstructed in MEGA7 (56). The negative slope and low *R*^2^ value indicate that time does not explain root-to-tip distances, measured in substitutions per site. (B) A permutation test was conducted, where dates were randomly shuffled among the root-to-tip distances 1,000 times, and each time a linear regression was conducted. The observed correlation coefficient (*r* = to 0.2 [yellow line]), was plotted among the distribution of *r* values from the permutations. The observed *r* value (yellow line) is greater than only 19 of 1,000 values composing the distribution. Additionally, the negative *r* value indicates that the relationship is root-to-tip distance is not correlated with time. Download Figure S4, PDF file, 1 MB

Figure S5  Molecular clock analysis for genomes in the TEA clade, except for the hypermutator isolate (2000031055). (A) Linear regression analysis of root-to-tip distances extracted by Tempest (56) from a neighbor-joining tree reconstructed in MEGA7 (56). The nearly horizontal slope and weak correlation (low *R*^2^ value) indicates that time does not explain root-to-tip distances, measured as substitutions per site. (B) A permutation test was conducted, where dates were randomly shuffled among the root-to-tip distances 1,000 times, and each time a linear regression was conducted. The observed correlation coefficient (*r* = 0.03 [yellow line]) value, was plotted among the distribution of *r* values from the permutations. The observed *r* value (yellow line) is greater than 651 of 1,000 values composing the distribution, indicating that the correlation coefficient is no greater than expected by chance. Download Figure S5, PDF file, 0.1 MB

Figure S6  Molecular clock analysis using only parsimony informative SNPs for A clade (in group) genomes with at least one sister taxon dated within 5 years. (A) Neighbor-joining tree, including remaining taxa. (B) Linear regression analysis of root-to-tip distances extracted by Tempest (56) from a neighbor-joining tree reconstructed in MEGA7 (56). The negatively correlated slope indicates that time does not explain root-to-tip distances, measured as substitutions per site. (C) A permutation test was conducted, where dates were randomly shuffled among the root-to-tip distances 1,000 times, and each time a linear regression was conducted. The observed correlation coefficient (*r* = to 0.47 [yellow line]) value, was plotted among the distribution of *r* values from the permutations. The observed *r* value (yellow line) is greater than 22 of 1,000 values composing the distribution, indicating that the correlation coefficient is no greater than expected by chance. Download Figure S6, PDF file, 1.3 MB

Table S1  List of *B. anthracis* strains, genome accession numbers, and associated metadata. Table S1, XLSX file, 0.03 MB

Table S2  SNP character states for 11,989 SNPs across all 193 *B. anthracis* genomes. Table S2, XLSX file, 7 MB

Table S3  Branch assignments for all SNPs. Table S3, XLSX file, 0.3 MB

Table S4  SNP character states for the 376 SNPs within the Tsiankovskii subclade. Table S4, XLSX file, 0.04 MB
