# Supplementary figures and images for: A Bacillus anthracis Genome Sequence from the Sverdlovsk 1979 Autopsy Specimens
Source: mBio. 2016 Sep 27;7(5):e01501-16. doi: 10.1128/mBio.01501-16 (PMC5050339; doi:10.1128/mBio.01501-16)

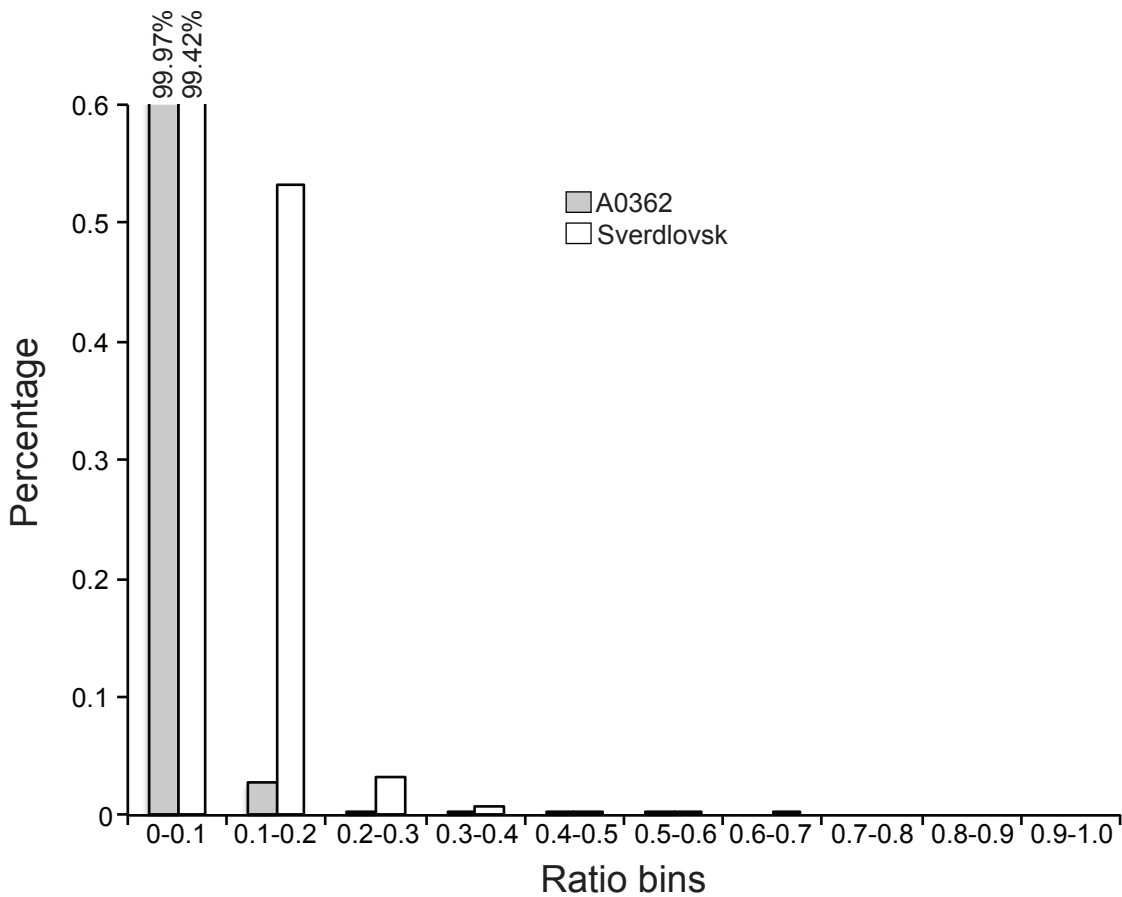

Supplement: Figure S1 — Read error rate profile across the genome for Sverd and a culture DNA (A0362: SRR2968203). Reads were aligned to the Ames ancestor, and the compositions of base calls were compared. Error rates were determined by dividing the number of minor allele calls by the total number of calls. The error rates were then binned into categories from no error to total error. The frequency of calls in each bin is represented by the height of histograms. The results demonstrate that while both genomes had low error rates, the Sverdlovsk genome had a higher error profile than a contemporary, pure culture. Download [file mbo005163008sf1.pdf]

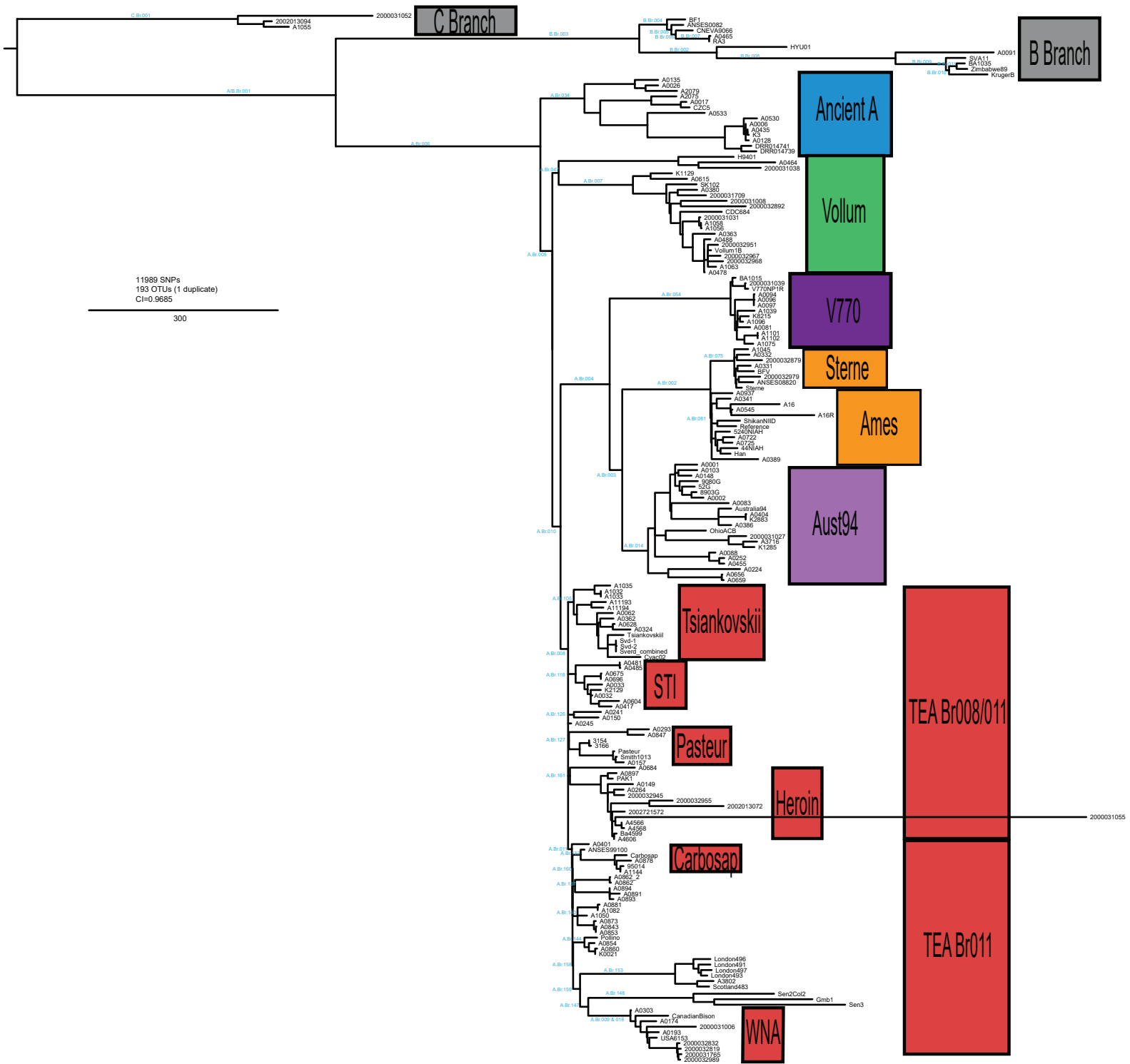

Supplement: Figure S2 — Maximum parsimony phylogeny of 193 B. anthracis genomes. Consistency index (CI [excluding parsimony uninformative characters]) = 0.9657. Names of major branches are indicated in blue text. Branch names within each clade are included in supplemental figure 3 with separate panels dedicated to each clade. Download [file mbo005163008sf2.pdf]

A. Ancient A

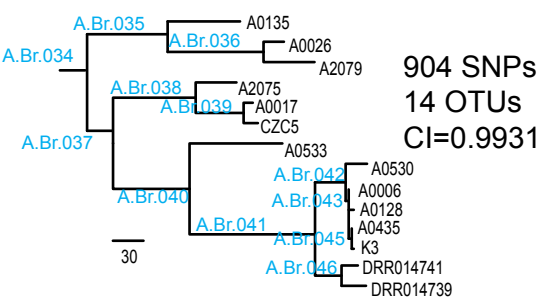

B. Vollum

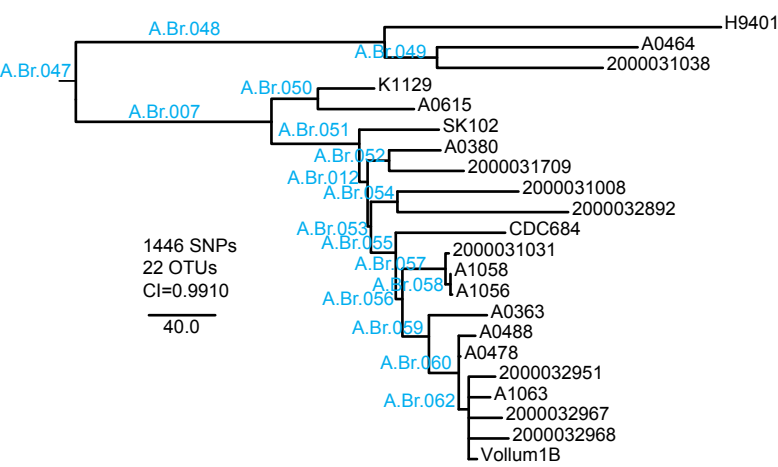

C. V770

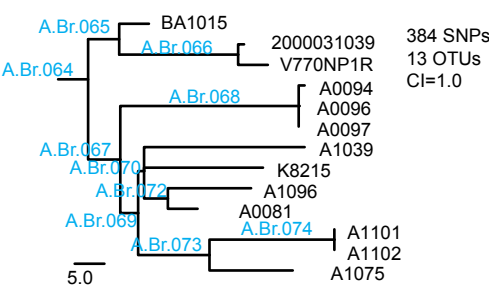

D. Sterne/Ames

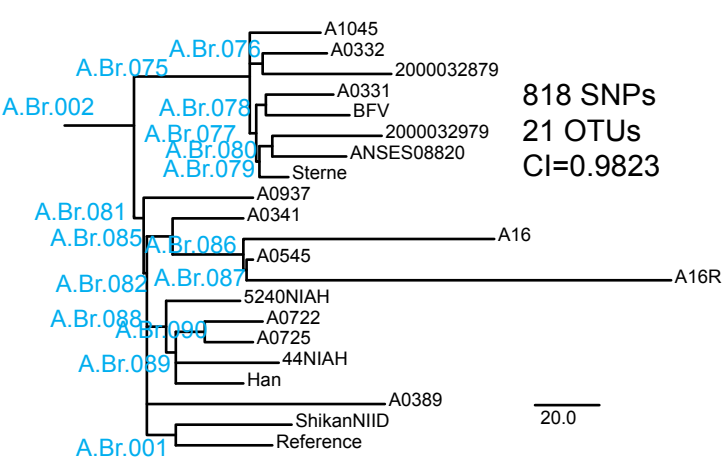

E. Australia94

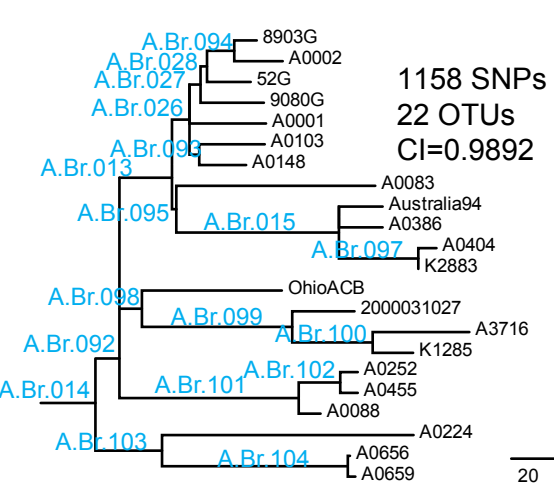

F. TEA

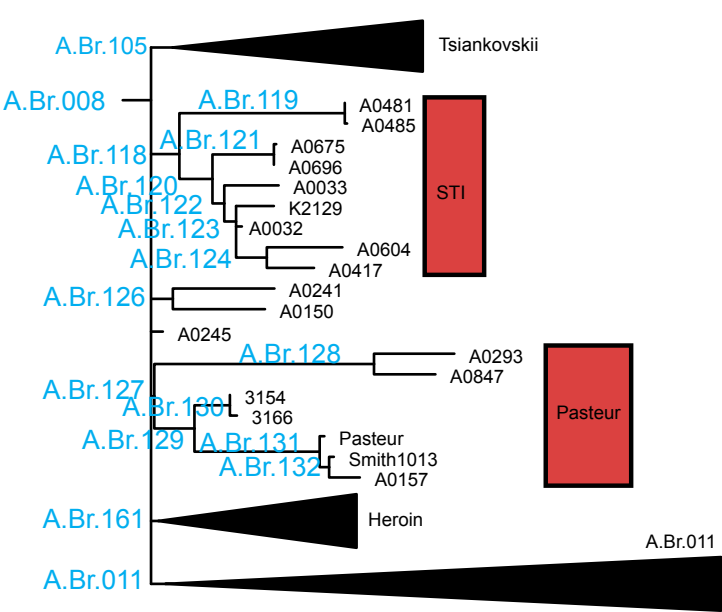

G. Tsiankovskii

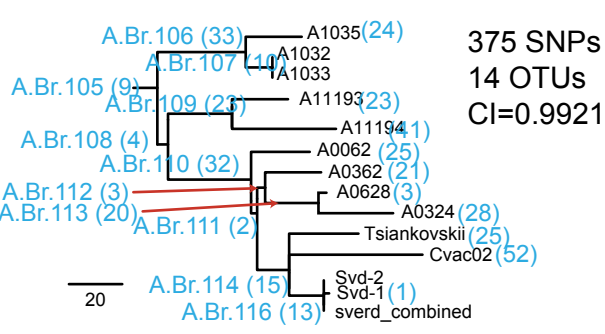

H. Heroin

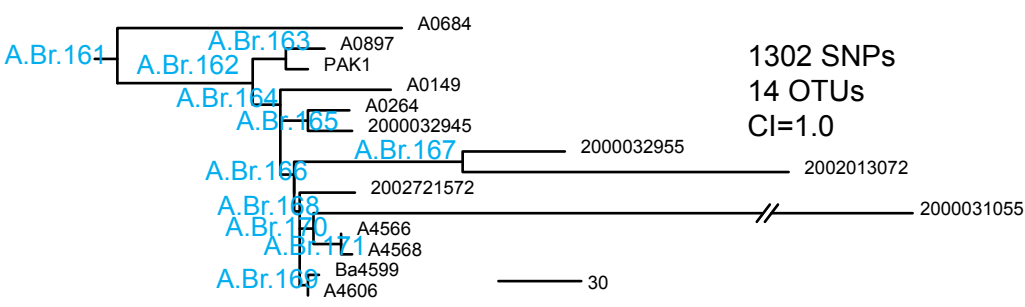

I. TEA 011

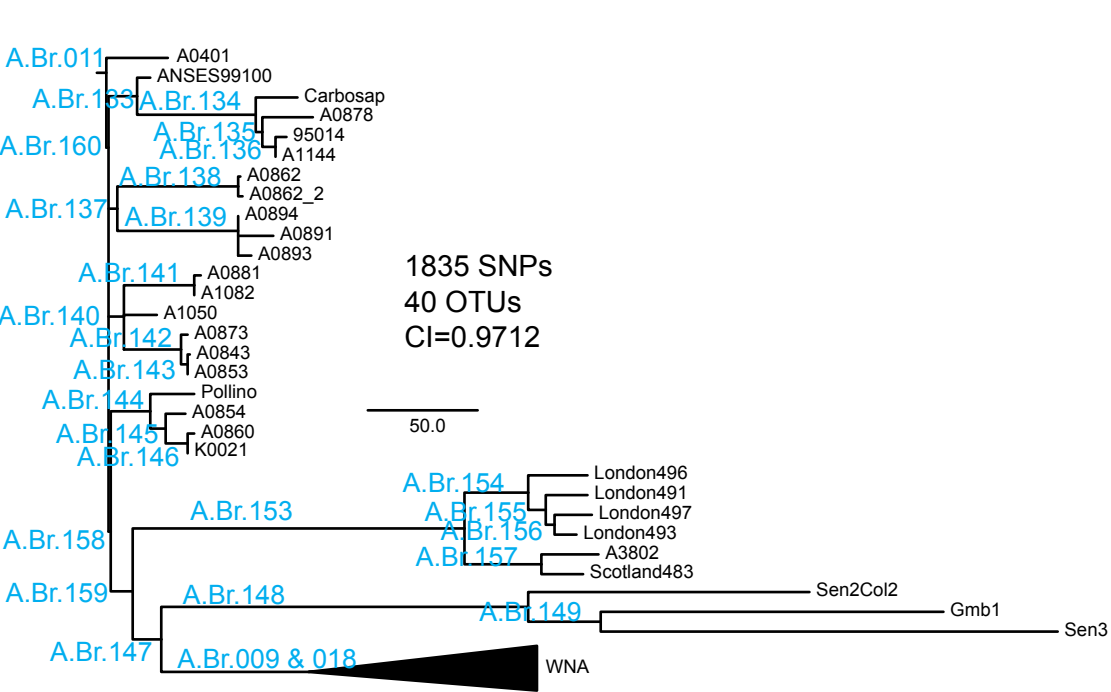

J. WNA

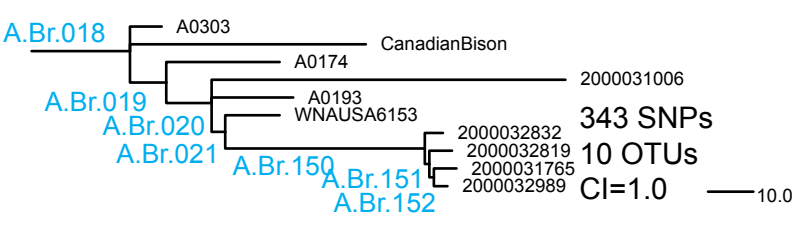

Supplement: Figure S3 — (A) Maximum parsimony phylogeny of the “Ancient A” clade. The formal name for this clade is A.Br.006/005. This clade currently contains 14 genomes and 904 SNPs. Consistency index (CI [excluding parsimony uninformative characters]) = 0.9931. Names of branches are indicated in blue text. (B) Maximum parsimony phylogeny of the “Vollum” clade. The formal name for this clade is A.Br.005/010. This clade currently contains 22 genomes and 1,446 SNPs. CI (excluding parsimony uninformative characters) = 0.9910. Names of branches are indicated in blue text. (C) Maximum parsimony phylogeny of the “V770” clade. The formal name for this clade is A.Br.004/003. This clade currently contains 13 genomes and 384 SNPs. CI (excluding parsimony uninformative characters) = 1.0. Names of branches are indicated in blue text. (D) Maximum parsimony phylogeny of the “Sterne/Ames” clade. The formal name for this clade is A.Br.003/014. This clade currently contains 21 genomes and 818 SNPs. CI (excluding parsimony uninformative characters) = 0.9823. Names of branches are indicated in blue text. (E) Maximum parsimony phylogeny of the “Australia94” clade. The formal name for this clade is A.Br.003/002. This clade currently contains 22 genomes and 1,158 SNPs. CI (excluding parsimony uninformative characters) = 0.9892. Names of branches are indicated in blue text. (F) Phylogeny of the “TEA” clade. This clade contains many large subclades that are presented in detail in panels G-I. Names of major branches are indicated in blue text. (G) Maximum parsimony phylogeny of the “Tsiankovskii” subclade (Fig. 3). This subclade is part of the “TEA” clade and is within the A.Br.008/011 clade. This subclade currently contains 14 genomes and 375 SNPs. CI (excluding parsimony uninformative characters) = 0.9921. Names of branches and branch lengths are indicated in blue text. (H) Maximum parsimony phylogeny of the “Heroin” subclade. This subclade is part of the “TEA” clade and is within the A.Br.008/011 clade. This [file mbo005163008sf3.pdf]

A

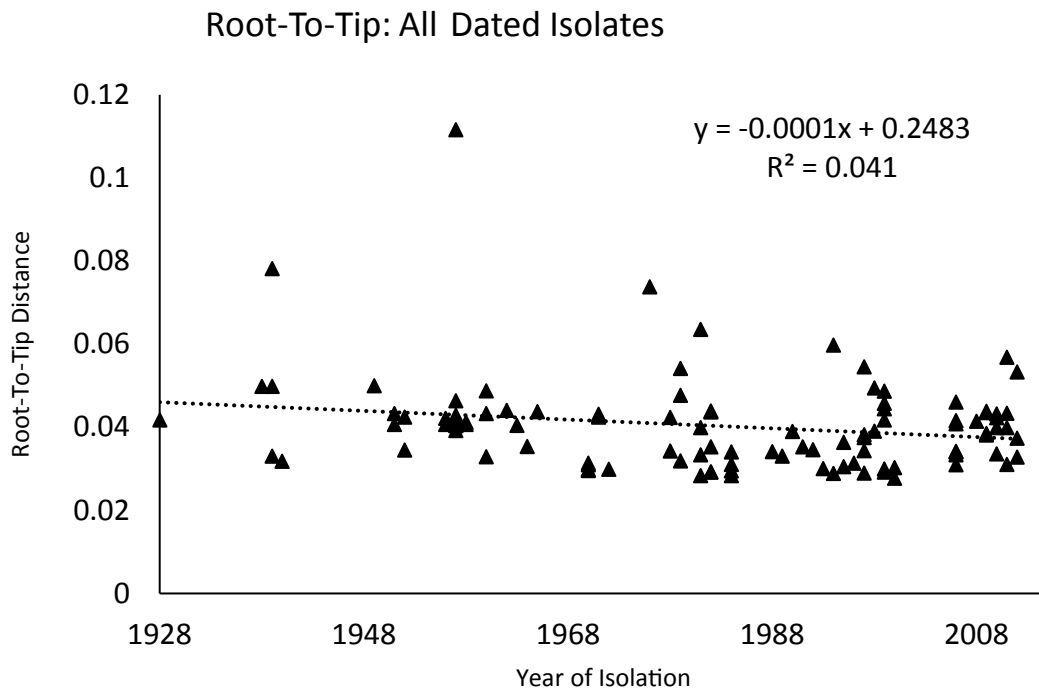

B

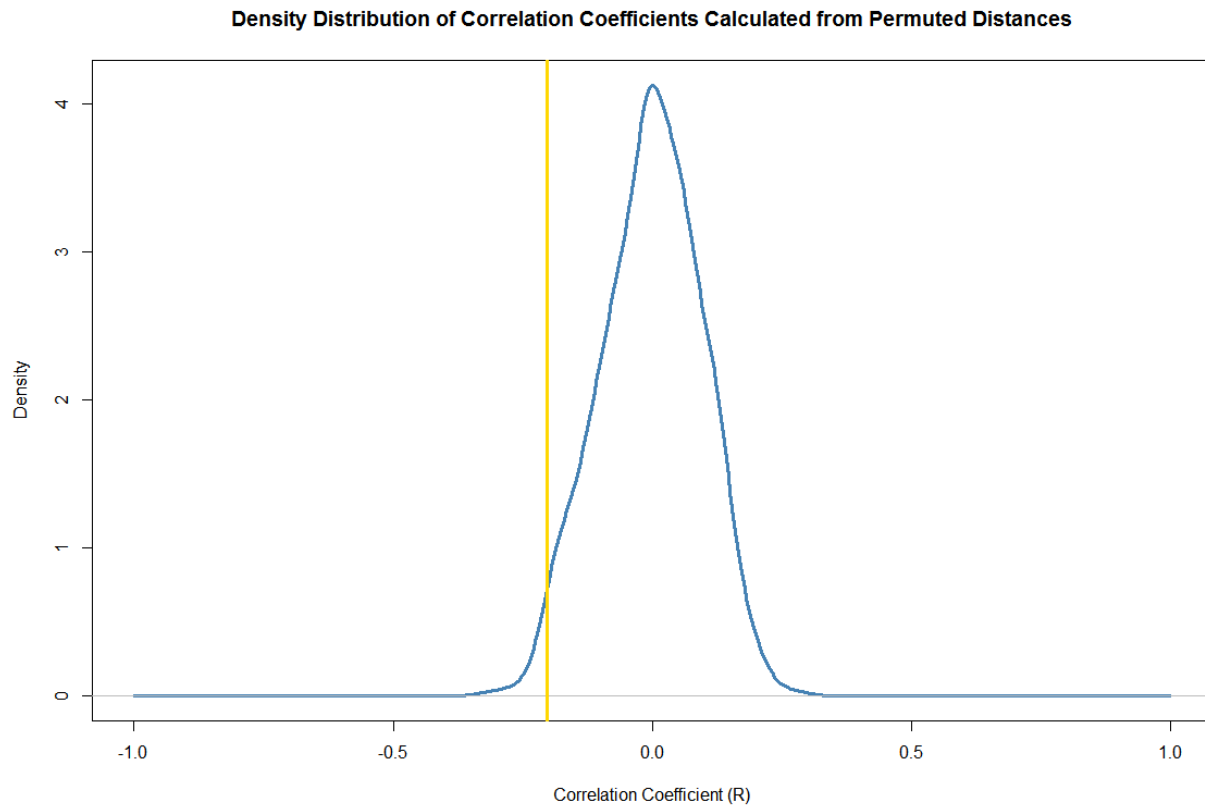

Supplement: Figure S4 — Molecular clock analysis for all genomes with isolation dates, except for the three C-branch isolates (2002013094, A1055, and 2000031052). (A) Linear regression analysis of root-to-tip distances extracted by Tempest (55) from a neighbor-joining tree reconstructed in MEGA7 (56). The negative slope and low R2 value indicate that time does not explain root-to-tip distances, measured in substitutions per site. (B) A permutation test was conducted, where dates were randomly shuffled among the root-to-tip distances 1,000 times, and each time a linear regression was conducted. The observed correlation coefficient (r = to 0.2 [yellow line]), was plotted among the distribution of r values from the permutations. The observed r value (yellow line) is greater than only 19 of 1,000 values composing the distribution. Additionally, the negative r value indicates that the relationship is root-to-tip distance is not correlated with time. Download [file mbo005163008sf4.pdf]

A

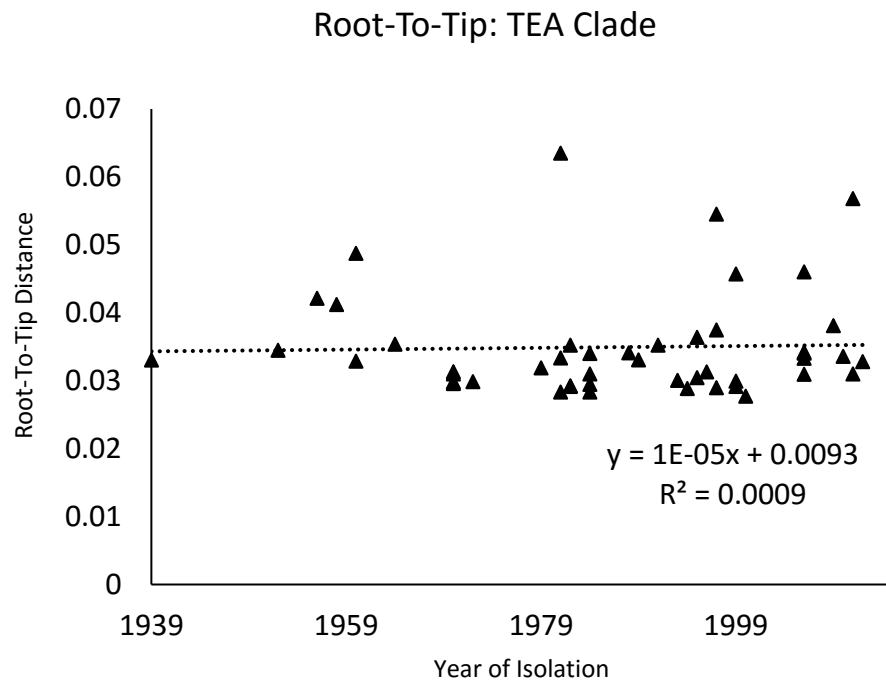

B

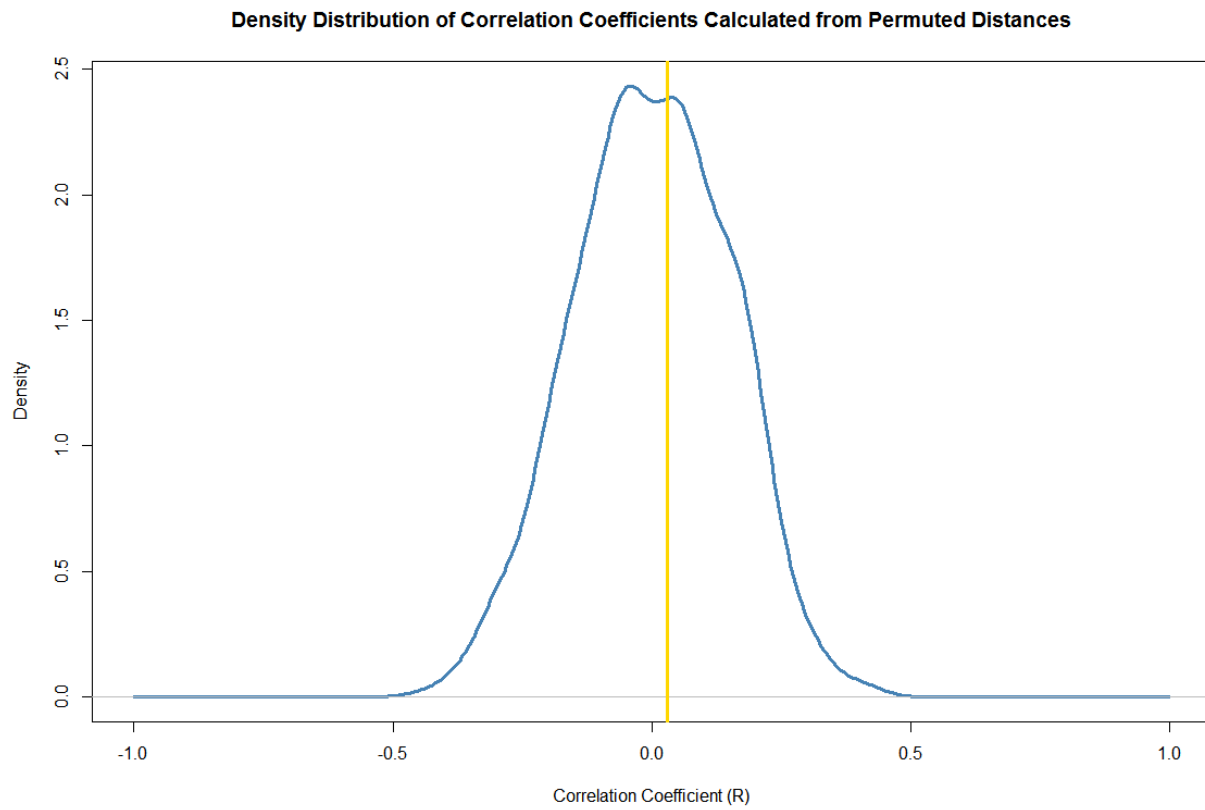

Supplement: Figure S5 — Molecular clock analysis for genomes in the TEA clade, except for the hypermutator isolate (2000031055). (A) Linear regression analysis of root-to-tip distances extracted by Tempest (56) from a neighbor-joining tree reconstructed in MEGA7 (56). The nearly horizontal slope and weak correlation (low R2 value) indicates that time does not explain root-to-tip distances, measured as substitutions per site. (B) A permutation test was conducted, where dates were randomly shuffled among the root-to-tip distances 1,000 times, and each time a linear regression was conducted. The observed correlation coefficient (r = 0.03 [yellow line]) value, was plotted among the distribution of r values from the permutations. The observed r value (yellow line) is greater than 651 of 1,000 values composing the distribution, indicating that the correlation coefficient is no greater than expected by chance. Download [file mbo005163008sf5.pdf]

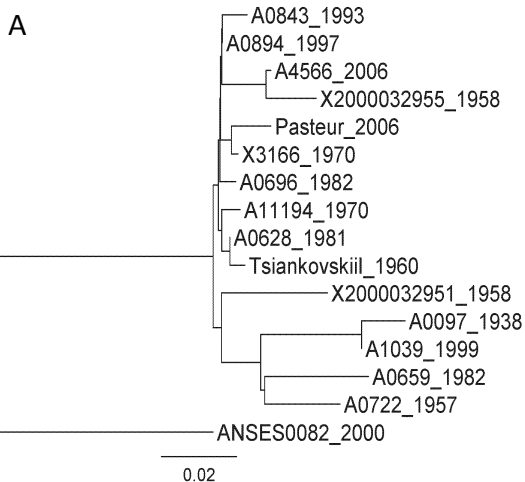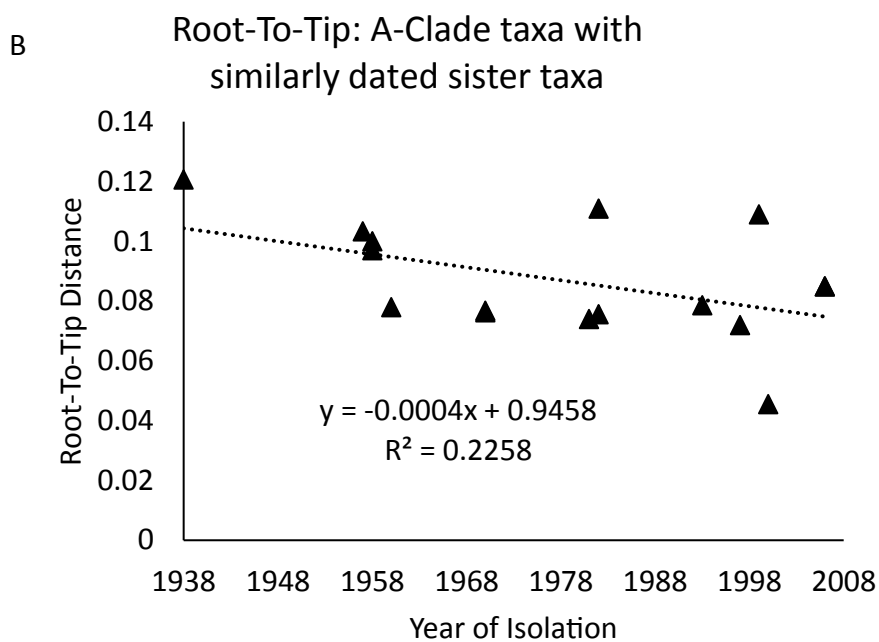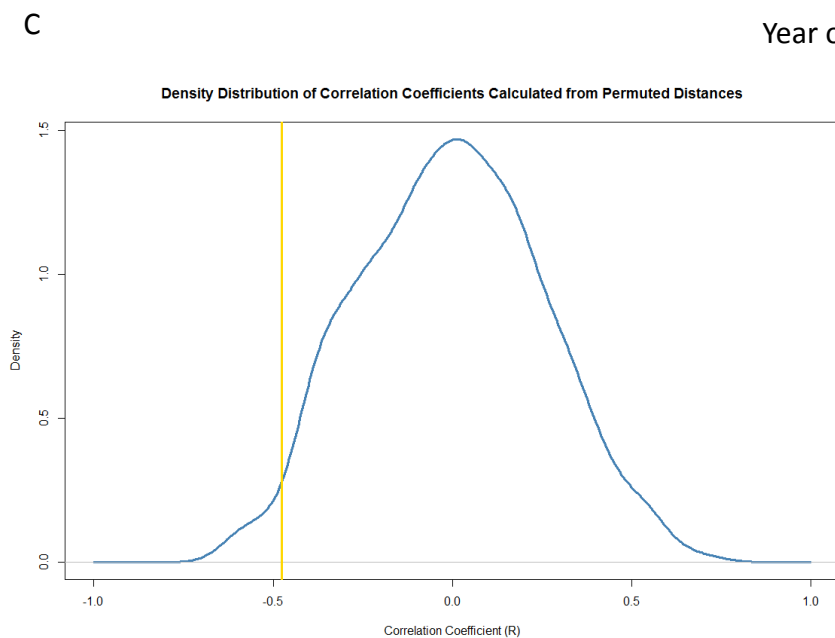

Supplement: Figure S6 — Molecular clock analysis using only parsimony informative SNPs for A clade (in group) genomes with at least one sister taxon dated within 5 years. (A) Neighbor-joining tree, including remaining taxa. (B) Linear regression analysis of root-to-tip distances extracted by Tempest (56) from a neighbor-joining tree reconstructed in MEGA7 (56). The negatively correlated slope indicates that time does not explain root-to-tip distances, measured as substitutions per site. (C) A permutation test was conducted, where dates were randomly shuffled among the root-to-tip distances 1,000 times, and each time a linear regression was conducted. The observed correlation coefficient (r = to 0.47 [yellow line]) value, was plotted among the distribution of r values from the permutations. The observed r value (yellow line) is greater than 22 of 1,000 values composing the distribution, indicating that the correlation coefficient is no greater than expected by chance. Download [file mbo005163008sf6.pdf]
